# A Novel Adverse Event Associated with Olaparib Therapy in a Patient with Metastatic Breast Cancer

**DOI:** 10.1155/2018/9529821

**Published:** 2018-06-27

**Authors:** Megan Wheelden, Leah Cream, Jeffrey Sivik, Mark Robson

**Affiliations:** ^1^Department of Medicine, Division of Hematology/Oncology, Penn State Hershey Medical Center, Hershey, PA, USA; ^2^Department of Pharmacy, Penn State Hershey Medical Center, Hershey, PA, USA; ^3^Department of Medical Oncology, Memorial Sloan Kettering Cancer Center, New York City, NY, USA

## Abstract

Olaparib was first FDA approved for use in women with advanced ovarian cancer and germline BRCA mutations. Based on the results of subsequent research, the use of this drug has been expanded to patients with metastatic breast cancer with germline BRCA mutation. With the use of a relatively new medication and a larger patient population eligible for therapy, monitoring for novel adverse events associated with therapy is important. This case represents a patient with metastatic breast cancer and germline BRCA2 mutation who developed erythema nodosum after initiation of therapy with olaparib capsules. Her characteristic rash appeared shortly after starting olaparib and recurred after restarting olaparib an additional two times. She was treated with short courses of prednisone therapy with or without holding olaparib with resolution of her rash. The patient was later restarted on olaparib capsules 200 mg twice daily, and she more recently has been maintained on olaparib tablets 300 mg twice daily. On both regimens, the patient experienced only attenuated episodes of erythema nodosum that have not required cessation of therapy or steroid therapy.

## 1. Introduction

Mutations of tumor suppressor breast cancer susceptibility genes 1 and 2 (BRCA1 and BRCA2) are well known to predispose affected individuals to the development of breast and/or ovarian cancers [1]. After laboratory studies demonstrated effective treatment of these mutated cells with poly(ADP ribose) polymerase (PARP) inhibitors, these medications were subsequently utilized in clinical trials [2]. Olaparib was first approved in 2014 for the treatment of patients with advanced ovarian cancer with a germline BRCA mutation with progression after at least three lines of chemotherapy [3]. This approval was based on the results of Study 19 published in the New England Journal of Medicine in 2012 [3, 4]. A logical extension of this successful trial was to investigate olaparib in the treatment of breast cancer. The recently published OlympiAD trial demonstrated a significant improvement in progression-free survival for patients with metastatic breast cancer and germline BRCA mutations treated with olaparib versus those who received standard therapy with capecitabine, eribulin, or vinorelbine [5]. With the expansion of the patient population eligible for olaparib therapy, monitoring for novel adverse events is critical. We present a case report of such an adverse event in a patient receiving olaparib therapy for metastatic breast cancer.

## 2. Case Presentation

A 45-year-old female with a history of metastatic breast cancer presented with an erythematous rash in her bilateral lower extremities. She was diagnosed approximately four years previously with estrogen and progesterone receptor positive, HER-2-negative breast cancer with involvement of twelve axillary lymph nodes. At the time of diagnosis, she was also found to have bony metastatic disease, and genetic testing revealed a deleterious 3036del4 germline BRCA2 mutation. After multiple lines of therapy, including most recently progressing on palbociclib and fulvestrant, the patient was switched to monotherapy with olaparib. Her rash began approximately three days after starting olaparib capsules at a dose of 300 mg twice daily. She subsequently developed progression of the erythematous nodules which became painful and limited her ambulation, bilateral lower extremity edema, fevers to 101.7°F (degrees Fahrenheit), and rigors. She tried diphenhydramine without any improvement in her symptoms, and patient then presented to the emergency department for evaluation. Her other home medications included levothyroxine, omeprazole, and cholecalciferol.

**Figure 1:**
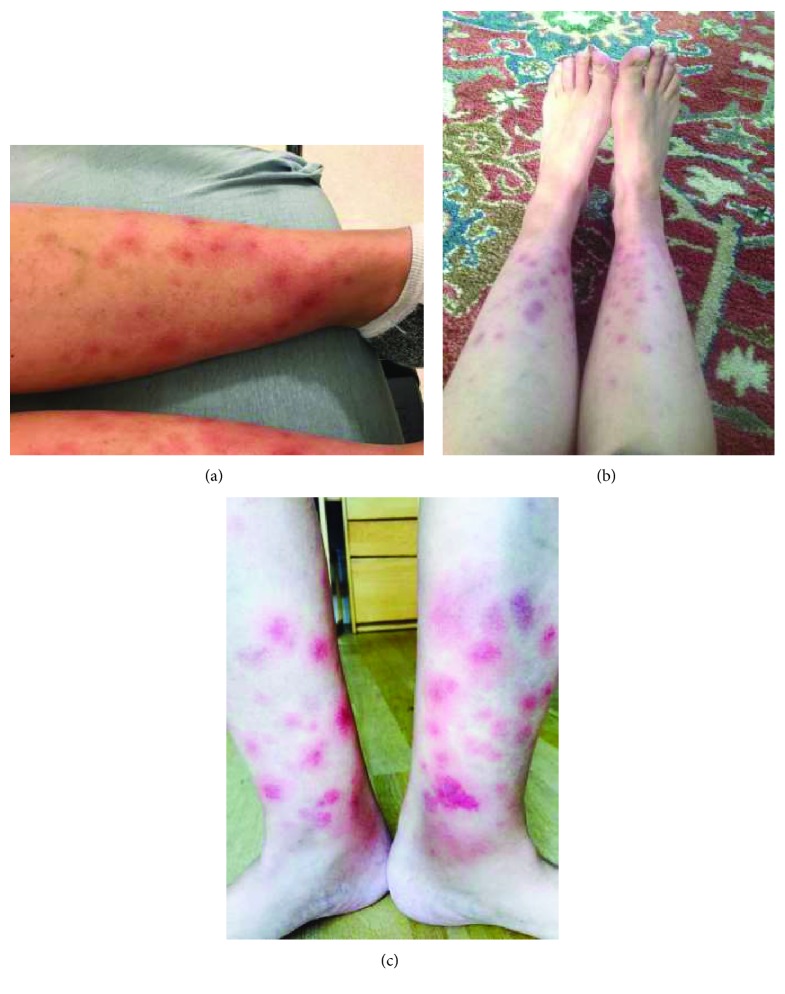
(a) Rash after initiation of olaparib therapy. (b) Recurrence of rash after the second course of treatment with olaparib. (c) Third recurrence of rash after reinitiation of olaparib.

Her vital signs were within normal limits. Her physical examination revealed multiple erythematous nodules over the bilateral distal lower extremities which were markedly tender to palpation, along with trace edema in her bilateral lower extremities (Figure 1(a)). Her basic metabolic profile was unremarkable, and her complete blood count demonstrated white blood count of 1.80 with absolute neutrophil count of 1200, hemoglobin of 11.2, and platelet count of 114. Her urinalysis was unremarkable, chest X-ray was normal, and blood cultures were sent. The patient was then admitted to inpatient Hematology-Oncology service for further evaluation of neutropenic fever. However, her infectious evaluation was unrevealing, and she then remained afebrile off antibiotics. Since this patient's symptoms and clinical examination were consistent with erythema nodosum, her olaparib was held. She was treated with nonsteroidal anti-inflammatory medications as well as acetaminophen as needed for ongoing pain. She was discharged with close outpatient follow-up. Her nodules improved dramatically within 24 hours of stopping olaparib and completely resolved within a week of withholding olaparib.

At her subsequent outpatient follow-up appointment, the patient was resumed on olaparib with a slow titration of dose to her prior regimen of 300 mg twice daily. The first day she took the 300 mg BID dose, the patient developed recurrence of her erythema nodosum, and she was started on a course of prednisone with a resolution of her symptoms (Figure 1(b)). The patient was then restarted on olaparib at a reduced dose of 250 mg twice daily, but afterwards developed erythema nodosum again (Figure 1(c)). Her symptoms resolved after completing another short course of prednisone therapy. At her next follow-up appointment, the patient was later resumed on olaparib capsules at a dose of 200 mg twice daily. She has tolerated this therapy at a reduced dose without any significant recurrence of erythema nodosum requiring cessation of olaparib or use of prednisone therapy. This patient had a complete metabolic response to treatment in the previously described metastatic bone lesions, and the previously described focal liver FDG avidity was no longer seen; in addition, her CA27.29 normalized. Once olaparib tablets were commercially available, she was subsequently changed to therapy with olaparib tablets 300 mg twice daily. Since then, she similarly has continued to have intermittent but attenuated episodes of erythema that have not required treatment or cessation of therapy.

## 3. Discussion

As previously noted, FDA approval of olaparib capsules for use in patients with ovarian cancer was based on the results of Study 19. Study 19 investigated olaparib as maintenance therapy in patients with high-grade serous ovarian cancer, and this study did not require assessment of BRCA1/2 germline mutations. Eligible patients had completed at least two prior platinum-based chemotherapy regimens with at least partial demonstrable response. The most frequent adverse events reported leading to either dose reduction or holding therapy were vomiting, nausea, and fatigue. The study mentioned one patient with the development of an erythematous rash necessitating cessation of olaparib therapy. However, this was deemed to be a grade 2 adverse event and no additional information regarding the rash was included in the published paper [4]. More recently, the SOLO2 study was published which examined the efficacy of the olaparib tablets for maintenance treatment for relapsed ovarian cancer patients with germline BRCA1 or BRCA2 mutations. Similar to what was evidenced in Study 19, the most common grade 3 adverse event was anemia—affecting 19% of those in the olaparib arm and 2% in the placebo arm. There were no reported adverse events related to skin manifestations in the study [6].

After the OlympiAD study's publication, olaparib was subsequently introduced into the treatment paradigm for metastatic breast cancer in patients with known germline BRCA medications. Olaparib's prior use in the treatment of relapsed ovarian cancer affords some insight regarding the potential adverse effects related to therapy [2]. However, given its extension to a metastatic BRCA-positive breast cancer patients, there remains a need for ongoing monitoring for adverse effects in this new patient population. In the OlympiAD study, anemia was cited as the most frequent reason for dose reduction in patients prescribed olaparib; the reported frequency of anemia was 40%, and dose reduction was necessitated in 13.7% of patients. The only adverse event with skin manifestations reported in the main body of the article was palmar-plantar erythrodysesthesia—present in 0.5% of patients in the olaparib group versus 20.9% of patients in the standard therapy group. However, in a review of the supplemental information for the OlympiAD study, one patient (0.5%) developed erythema nodosum requiring treatment discontinuation [5]. Therefore, our patient was unique in regard to continuing olaparib therapy after the development of erythema nodosum.

Erythema nodosum is well described as the most common form of an uncommon condition—panniculitis or inflammation of the subcutaneous fat [7]. The characteristic presentation of erythema nodosum is the onset of painful, erythematous nodules in the skin and subcutaneous tissues. These lesions are also typically elevated, approximately 1 to 6 cm in diameter, and distributed throughout the bilateral lower extremities [8]. Patients frequently have associated constitutional symptoms—including fever, fatigue, and malaise—and can also experience myalgia, headache, and abdominal pain [9]. Current practice guidelines do not necessitate a biopsy to confirm the diagnosis in patients with classic presentations [7].

There is currently no consensus for treatment of erythema nodosum, largely due to the uncommon nature of the condition. The majority of cases are self-limited, and patients are provided with symptomatic management [7]. It is reported that the majority of cases of erythema nodosum resolve within a few weeks—ranging from 3 to 4 weeks to up to 6 weeks for severe cases [8, 9]. The role for treatment with medications such as nonsteroidal anti-inflammatory medications, potassium iodine therapy, or corticosteroids is based on the reports of case reports or series [8, 10–12].

Erythema nodosum is categorized as a hypersensitivity response, and this can be as a response to a significant variety of stimuli [9]. Although the exact immune-mediated mechanism has yet to be clearly elucidated, it has been postulated to be due to a delayed hypersensitivity response [7]. A 1998 French study reviewing the charts of 129 patients with confirmed erythema nodosum demonstrated that 55% of cases were idiopathic, and of the etiologies identified, the most common were streptococcal infections (28%) and sarcoidosis (11%) [13]. In more recent years, drugs have emerged as a common cause of erythema nodosum, with an extensive list of causative agents based on case reports—with more longstanding known offending agents including sulfonamide antibiotics and oral contraceptives [9].

Interestingly, the patient in our case report developed erythema nodosum on both the olaparib tablet and capsule preparations, although her most severe episodes were on the olaparib capsules at a dose of 300 mg twice daily. In a review of the prescribing information, the inactive ingredients for olaparib capsules are lauroyl polyoxylglycerides, hypromellose, titanium dioxide, gellan gum, potassium acetate, shellac, and ferrosoferric oxide [14]. The inactive ingredients for olaparib tablets include copovidone, mannitol, colloidal silicon dioxide, sodium stearyl fumarate, hypromellose, polyethylene glycol 400, titanium dioxide, and ferric oxide yellow for all dose strengths. In addition to what is previously listed, the 150 mg olaparib tablets also contain ferrosoferric oxide [15]. In a review of the pharmacokinetics, the bioavailability of the tablets is higher than capsules, and it was reported that the area under the curve (AUC) for people taking 300 mg tablets twice daily was 77% higher when compared to those taking 400 mg capsules twice daily [15]. Finally, both preparations have different half-lives, with the mean half-life of the capsules and tablets 11.0 and 14.9, respectively [14, 15]. A literature search did not reveal any articles associating the inactive ingredients in both preparations—including hypromellose, gellan gum, potassium acetate, lauroyl polyoxylglycerides, and shellac—with erythema nodosum.

In a review of the current literature, this is among the first reported cases of erythema nodosum due to olaparib therapy and the first after the publication of the OlympiAD study. Although dose is not usually related to erythema nodosum, it seemed to in this case as the patient's erythema nodosum was most pronounced and symptomatic on olaparib capsules at a dose of 300 mg twice daily compared to 200 mg twice daily. There may be a dose-related off target effect of PARP inhibition on inflammation which warrants more investigation. In addition, the episodes occurred with travel and stasis. She continued to have attenuated episodes of erythema nodosum on olaparib capsules at 200 mg twice daily as well as olaparib tablets 300 mg twice daily. Given the higher bioavailability and longer half-life of the tablets compared to capsule preparations, it is interesting that her skin findings and symptoms were less severe on the tablets 300 mg twice daily. However, it is possible that this is due to the attenuation of the underlying hypersensitivity response over time.

## 4. Conclusion

The use of olaparib has only recently been incorporated into treatment strategies for patients with germline BRCA1/2 mutations with metastatic breast cancer. Although olaparib was FDA approved for use in patients with BRCA mutations and advanced ovarian carcinoma since December 2014, this case represents the second reported patient developing erythema nodosum while taking olaparib [3, 5]. Her symptoms, physical examination, and recurrence of rash with reintroduction of olaparib on multiple occasions support the diagnosis of erythema nodosum related to olaparib therapy. This case highlights the importance of monitoring for previously undescribed adverse events while on novel therapies. Additionally, it also provides a strategy for managing erythema nodosum related to olaparib therapy that allows for reintroduction and maintenance with dose-reduced olaparib after such an event.

## References

[1] Rizvi W., Truong P., Truong Q. (2017). Metastatic breast cancer with BRCA mutation discovered by next-generation sequencing responding to olaparib. *Cureus*.

[2] George A., Banerjee S., Kaye S. (2017). Olaparib and somatic BRCA mutations. *Oncotarget*.

[3] Kim G., Ison G., McKee A. E. (2015). FDA approval summary: Olaparib monotherapy in patients with deleterious germline *BRCA*-mutated advanced ovarian cancer treated with three or more lines of chemotherapy. *Clinical Cancer Research*.

[4] Ledermann J., Harter P., Gourley C. (2012). Olaparib maintenance therapy in platinum-sensitive relapsed ovarian cancer. *The New England Journal of Medicine*.

[5] Robson M., Im S.-A., Senkus E. (2017). Olaparib for metastatic breast cancer in patients with germline *BRCA* mutation. *The New England Journal of Medicine*.

[6] Pujade-Lauraine E., Ledermann J. A., Selle F. (2017). Olaparib tablets as maintenance therapy in patients with platinum-sensitive, relapsed ovarian cancer and a *BRCA1/2* mutation (SOLO2/ENGOT-Ov21): a double-blind, randomised, placebo-controlled, phase 3 trial. *The Lancet Oncology*.

[7] Blake T., Manahan M., Rodins K. (2014). Erythema nodosum – a review of an uncommon panniculitis. *Dermatology Online Journal*.

[8] Mana J., Marcoval J. (2007). Erythema nodosum. *Clinics in Dermatology*.

[9] Requena L., SanchezYus E. (2007). Erythema nodosum. *Dermatologic Clinics*.

[10] Friedman E. S., LaNatra N., Stiller M. J. (2002). NSAIDs in dermatologic therapy: review and preview. *Journal of Cutaneous Medicine and Surgery*.

[11] Barr W. G., Robinson J. A. (1981). Chronic erythema nodosum treated with indomethacin. *Annals of Internal Medicine*.

[12] Mert A., Kumbasar H., Ozaras R. (2007). Erythema nodosum: an evaluation of 100 cases. *Clinical and Experimental Rheumatology*.

[13] Cribier B., Caille A., Heid E., Grosshans E. (1998). Erythema nodosum and associated diseases. A study of 129 cases. *International Journal of Dermatology*.

[14] US Food and Drug Administration (2014). Highlights of prescribing information, Reference ID: 3675412. https://www.accessdata.fda.gov/drugsatfda_docs/label/2014/206162lbl.pdf.

[15] US Food and Drug Administration (2014). Highlights of prescribing information, Reference ID: 4206580. https://www.accessdata.fda.gov/drugsatfda_docs/label/2018/208558s001lbl.pdf.

